# Transactions of the Ohio Medical Society

**Published:** 1879-04

**Authors:** 


*Transactions of the Ohio Medical Society.* This is a sprightly little volume of 227 pages, containing many valuable instructive papers and doing much credit to the profession of Ohio.

